# A decline in perceived social status leads to post-traumatic stress disorder symptoms in adults half a year after the outbreak of the COVID-19 pandemic: consideration of the mediation effect of perceived vulnerability to disease

**DOI:** 10.3389/fpsyt.2023.1217264

**Published:** 2023-07-21

**Authors:** Yean Wang, Shuge Xu, Yue Chen, Haijuan Liu

**Affiliations:** ^1^School of Social Development and Public Policy, Beijing Normal University, Beijing, China; ^2^School of Sociology, Wuhan University, Wuhan, China; ^3^School of Sociology, Central China Normal University, Wuhan, China

**Keywords:** COVID-19, perceived social status, post-traumatic stress disorder, perceived vulnerability to disease, Hubei China, propensity score matching

## Abstract

**Background/purpose:**

In December 2019, Wuhan, Hubei, China firstly reported the existence of the COVID-19 virus. It is crucial to prioritize the psychological well-being of citizens in lockdown cities and make more strides in the academic field of post-traumatic stress disorder (PTSD) to prepare for the post-pandemic era.

**Methods:**

We took the cognitive-relational theory as our basis and collected Hubei province-level data (*N* = 3,465) to examine the impact of perceived social status decline on the prevalence of PTSD symptoms, and checked the mediating effect of perceived vulnerability to disease (PVD) during the period of psychological adjustment.

**Results:**

Using propensity score matching, we estimate the average treatment effect of perceived social status decline on PTSD level, and we robustly regress the two with weight adjustment generated in matching. We found that more decline in perceived social status is associated with a worse degree of PTSD symptoms, and confirmed PVD’s buffering role although the mediating effect was not as high as hypothesized.

**Conclusion and implications:**

Our study confirmed the decisive role of subject social status in health prediction compared to traditional socioeconomic measures, which extends the cognitive-relational in examining socioeconomic status and contributes to the dialog on socioeconomic inequality. We also suggested providing more social support at the community level and enhancing individuals’ positive understanding to protect mental health.

## 1. Introduction

A large amount of research evidence has revealed the prevalence of various psychological illnesses and related symptoms, including depression, anxiety, insomnia, and post-traumatic stress disorder (PTSD) since the outbreak of the COVID-19 pandemic (1–3). Since the COVID-19 pandemic, as a public health emergency, has negatively affected the life of whole people around the world, it is qualified as a traumatic event, exposure to which is the prior cause of the development of PTSD (4, 5). A higher combined prevalence of post-pandemic PTSD (~23%) than the estimated pooled prevalence after other disasters, such as major traumatic events (~20%) and floods (~16%), has been discovered, indicating that it is common for people who experienced an infectious disease outbreak to develop PTSD (3). Therefore, more research efforts should be made in the area of post-COVID-19 PTSD to assist in obtaining a thorough understanding of the detrimental impact of the pandemic.

Various COVID-19 studies have covered the negative effects of many pandemic-related traumatic experiences on PTSD and related responses. Research focuses can be concluded as follows: personal (i.e., sleep quality, experience or history of physical or psychological comorbidity), infectious-related factors (i.e., exposure, perceived vulnerability to disease), and societal factors [i.e., social isolation, stigmatization and discrimination, and social status decline; (3, 6)]. Among these factors, social status decline is one of the most important points that has attracted much attention from researchers. Large-scale pandemics have the potential to greatly increase global morbidity and mortality and cause profound disruptions in economic, societal, and political statuses (3). From the macro perspective, social distancing measures lead to the suspension of production and multiple working activities. For individual employees and their families, temporary layoffs of work during quarantine generate insecurity in employment and even financial loss when working part-time. Job changes that occur during quarantine put people under huge stress and financial strain, negatively impacting their quality of life and social standing (7). Those who are self-employed or are unable to work remotely while in quarantine may suffer more severe socioeconomic distress, which could affect how they perceive their social status. However, whether the perceived social status decline will influence the prevalence of PTSD or related traumatic stress symptoms has not been studied systematically.

Both cognitive-relational theory (8) and the cognitive model of PTSD (9) emphasize the importance of subjective appraisal of a traumatic event when assessing the psychological impact of a trauma or stress. When a threat is perceived to be more severe than it actually is, one may experience increased psychological stress that could develop into PTSD. Therefore, based on the theoretical background, we decided to explore the effect of perceived social status decline on developing PTSD symptoms. In addition, high perceived vulnerability to disease during an infectious disease outbreak can also contribute to the formation of PTSD by inducing traumatic stress responses (10). As the theory of fundamental social causes states, socioeconomic status (SES) is especially related to one’s perceived control over life (11). Low SES is characterized by the perception that one’s actions are persistently influenced by external forces that are beyond one’s individual control and influence. When perceiving social status decreases during the pandemic, individuals’ sense of control over life decreases accordingly. When losing control over their life, individuals can feel vulnerable to the threat of infectious disease since they have limited resources to protect or support themselves through difficult times. Therefore, the mediating effect of perceived vulnerability to the disease on the relationship between perceived social status decline and PTSD symptoms deserves more extensive investigation.

Although the negative influences of the COVID-19 pandemic on the social and psychological well-being of Chinese, especially Hubei residents, had been investigated by some researchers at the beginning of the first outbreak, there has been little attention to study how—the detrimental consequences on the social aspect will influence PTSD symptoms under the traumatic public health crisis. Additionally, since PTSD is a psychological disorder that occurs in a period after a traumatic event, the study aiming to examine the PTSD level of Hubei residents needs to be conducted in the post-pandemic time. However, to our knowledge, none of the studies focusing on the COVID-19 pandemic had empirically investigated the PTSD level of Hubei residents in the post-pandemic era. Therefore, to fill these research gaps mentioned above and to improve understanding of the social and psychological consequences of infectious disease outbreaks, this study was conducted on the adult population of the Hubei province of China approximately half a year after the first COVID-19 outbreak to measure whether perceived social status decrease would cause the incidence of PTSD reactions in people and the mediating effect of perceived vulnerability to disease. The existing knowledge of PTSD and its related factors due to global infectious disease outbreaks will be discussed first, and then the findings of the current study will be presented.

## 2. Literature review

### 2.1. PTSD and infectious disease outbreaks

PTSD refers to a stress-related mental disease that affects persons who have encountered or experienced a life-threatening traumatic incident, placing considerable strain on individuals and society (12). Various chronic symptoms have been known to arise from the development of PTSD, such as intrusive memories and trauma re-experiencing through flashback-like dissociative reactions, the desire to avoid trauma-related thoughts, feelings, places, or people, emotional numbing or continuously negative cognition and mood, and hyperarousal, such as trouble sleeping, anxiety, and irritability (13, 14). Although not everyone who experiences traumatic stress will develop PTSD, it will be difficult for those who are diagnosed with persistent PTSD to recover completely or receive treatment. Failed recovery from PTSD can have long-term harmful effects on an individual’s social function, family life, and personal health and may cause financial burdens (15).

Previous literature has discovered that the direct cause of PTSD is exposure to traumatic events (5, 16). As a public health emergency closely related to all people, COVID-19 has been confirmed as a qualified traumatic event that can lead to PTSD symptoms in the general population (4). Studies on the relationship between infectious disease outbreaks and people’s mental health found that post-traumatic stress (PTS) is common in those who encounter infectious disease outbreaks [ex. SARS, Ebola, H1N1, etc.; (17)]. Therefore, due to the enormous detrimental consequences of PTSD on individuals and their families, investigating the prevalence of PTSD or PTS symptoms in the post-pandemic period is of great importance in understanding the psychological burden on the public and possible identification and intervention strategies for reducing the negative effects of the trauma brought by the pandemic.

### 2.2. Risk factors for post-pandemic PTSD

Various studies have investigated the impact of the pandemic on individuals’ mental well-being. Pandemic outbreaks that lead to worldwide detrimental consequences can be classified as traumatic events that could contribute to the development of PTSD (4). Pandemic-related stressful experiences, like quarantine, infection of self or family or friends, and potential financial loss, are all traumatic incidents that play as factors in the development of PTSD symptoms in individuals. According to existing studies, predicting risk factors for post-pandemic PTSD after infectious disease outbreaks can be classified into several aspects: personal, infectious-related, and social factors (3, 6).

The first personal factor that could lead to PTSD symptoms is sleep disruption. One of the serious health problems brought about by quarantine that could promote PTSD formation is irregular sleep schedules or even insomnia (3). With the suspension of school or business activities, people’s regular schedules are disrupted, affecting the quantity and quality of their sleep (18). Poor sleep quality during quarantine has been shown to be a strong predictor and a vital characteristic of PTSD (14, 19). During the immediate aftermath of trauma, subjective sleep problems and interruption of REM sleep can indicate future PTSD development (14). Second, people with physical comorbidities have been proven to have a higher risk of developing PTSD (3). A study conducted after the SARS pandemic proved that the presence of chronic medical illnesses diagnosed before the onset of the pandemic and avascular necrosis were independent predictors of post-pandemic PTSD (20). At the same time, patients with comorbid diseases or psychiatric disorders were also found to be more susceptible to PTSD (3).

One of the other significant focuses of preexisting studies is infectious-related factors, including exposure to COVID-19 (both disease exposure and informational exposure) and perceived vulnerability to disease. In regards to exposure to disease, both previous studies on the SARS epidemic (16, 21) and recent research on the COVID-19 pandemic reveal the high rate of PTSD or PTS symptoms in frontline healthcare workers who have been constantly exposed to infectious disease patients in their workplaces (22–24). With the shortage of personal protective equipment plus the overloaded work intensity and often extended duration of shifts, frontline medical workers and health care providers continued to be exposed to extreme worry about personal safety and unavoidable emotional shock that is caused by the demise of infected patients (3). In addition, the level of exposure to pandemic-related information and news also contributes to the formation of PTSD or PTS reactions. When being bombarded with mass negative information regarding the pandemic, individuals’ psychological conditions are more likely to be harmed drastically (3). The public, under a state of panic and worry due to the newly discovered virus, was more subjected to the influences of explosive fake news and posts regarding transmission mechanisms of the disease and infection-prevention techniques, which could result in more stress and anxiety regarding the pandemic outbreak and increase the possibility of PTSD (25).

Furthermore, perceived vulnerability to disease or perceived risk of infection also has a positive relationship with the prevalence of PTSD symptoms (3, 26). Individuals who perceive themselves as highly likely to be infected may view this pandemic as more personally life-threatening and experience more traumatic stress than people who consider themselves less susceptible to COVID-19 (26).

Moreover, pandemic literature also strived to study social factors of PTSD, including social isolation and stigmatization, and discrimination. Social isolation is a major stressor activating psychological and physiological stress responses (27) and is an effective indicator of traumatic stress during life-threatening infectious disease outbreaks (28). Given the expanding COVID-19 crisis, policymakers in numerous nations hastily adopted social distancing and quarantine policies. Although quarantine effectively assists in controlling the spread of disease, confining individuals’ freedom to go out or meet other people as usual increases the risk of mental illness and the prevalence of psychological distress symptoms (29). A meta-analysis conducted by Yuan et al. (3) concluded that the pooled prevalence of post-pandemic PTSD among pandemic victims who experienced quarantine during the outbreak (15%) was higher than that among victims without quarantine experience (5%). In addition, among people who experienced quarantine, as the length of confinement increases, the rate of stress in individuals increases accordingly (30). In addition, the experience of stigmatization and discrimination is another social factor that predicts post-pandemic PTSD in individuals. Many people claimed being discriminated against due to where they came from or lived during the disease outbreak or whether they had been infected or had close contact with confirmed cases (3).

### 2.3. Theoretical construction and hypothesis

#### 2.3.1. Social status decline and PTSD

The traumatic experiences of declines in social status due to the pandemic, relevant financial loss and job instability as a result of quarantine created serious socioeconomic distress. It was a risk factor for symptoms of psychological disorders, including PTSD (31). Typically, social status is assessed through income, level of education, and employment (32). In addition to household income and educational attainment, employment is one of the other important objective and quantifiable indicators of individual social status in general (11). Employment not only indicates human capital but also has strong predictive validity in the material capital of individuals since it is typically closely related to the economic status of individuals. The COVID-19 pandemic has drastically affected socioeconomic development and work activities worldwide. Although the effects of COVID-19 on the economy at the macro and micro levels are still challenging to determine, the influences on the people and the families of those who lost their jobs, suffered temporary layoffs, or kept their jobs but faced the loss or worsening of their working situations have been analyzed by researchers (30). Nonetheless, apart from the impact of the objective decline in social status, how individuals perceive their changes in social status could have more detrimental effects on their psychological well-being.

#### 2.3.2. Transactional model of stress and perceived social status decline

The transactional model of stress and coping (a.k.a. cognitive-relational theory) is a theoretical model that has been applied to understand the effects of stress in numerous studies (8). It was then adapted to explain PTSD by Kleber, Brom, and Defares (33). It played a fundamental role in developing an etiological model evaluating the influence of stress and coping strategies on psychological outcomes during stressful events. It is outstanding in that it focuses on the effect of individuals’ cognitive assessment of trauma on their stress level, which indicates the impact of a significant interacting variable besides the traumatic event itself in forming PTSD (34). According to the transactional model of stress, subjective perceptions of threat may not always match the level of threat indicated by more objective measures and circumstances in life, and perceptions of threat may be more essential in determining levels of distress. Only when individuals perceive an event as stressful can it be such.

The transactional model of stress suggests the process of determining the importance of events for oneself (35). The primary appraisal includes assessments of events and interactions as threats or challenges or as being fundamental to oneself and entails determining the significance of a transaction for one’s health. Threat appraisals considers the possibility of future harm or loss, both of which have detrimental effects. However, challenge appraisals focus on the positive interpretations of events and represent the expectation of progress or gain from experience. Individuals with high levels of negative affectivity were more likely to appraise events as threatening, while those with low levels of negative affectivity appraised them as a challenge (36, 37). In the context of global public health crises, such as the COVID-19 pandemic, various traumatic and stressful experiences, such as exposure to infection, social isolation, housing instability, and loss of control over social or financial status, all contribute to an increase in negative affectivity in public in general. Due to various uncertainties regarding transmission, treatment, and health impacts of COVID-19 at the beginning and the huge population density of China, which could speed up virus spread and medical system breakdown, Chinese people, especially Hubei residents, could be more anxious during the first outbreak comparing to people who lived in other countries that were affected later. Under these circumstances, it is highly possible that Hubei residents possessed an increased level of negative affectivity that led to threat appraisals. Therefore, with higher negative affectivity generated in the pandemic, individuals are more likely to appraise their job and financial instability as a solid threat. The results of studies have demonstrated a strong relationship between threat appraisal and coping strategies, which might further contribute to improper adaptation to stressful situations and increase psychological suffering (35).

Meanwhile, a growing amount of studies have shown that subjective ideas about one’s social status are a better predictor of mental health outcomes than objective measures such as educational level, income, and occupation (38, 39). Job insecurity is defined as “the perceived threat of job loss and the worries related to that threat” (40). It is a subjective anticipatory perception, with worry and fears about the future of one’s current job in the short or medium term (41). The current COVID-19 literature has revealed that the perceived risk of both employment and financial threat have negative effects on the physical, psychological, and psychosocial well-being of people (41). It has been demonstrated that greater employment insecurity and job loss have been linked to greater depression symptoms since the start of the pandemic (7, 42). Additionally, individuals who believed that their work situation will worsen after the quarantine demonstrated higher perceived stress (30). It has also been shown (43) that workers perceive a loss of control in times of economic turbulence (such as significant crises and recessions, such as the one brought on by the COVID-19 pandemic), making the negative effects of job insecurity on mental health even worse (44). Nevertheless, few investigations have been conducted on the influence of subjective social status on PTSD.

Therefore, to solve the research puzzle of how the perception of a decrease in self-perceived social status influences PTSD symptom development, we established our first hypothesis:

*Hypothesis 1:* A decline in perceived social status contributes to the prevalence of PTSD symptoms in people who lived in Hubei Province, China, during the outbreak of the COVID-19 pandemic at the beginning of 2020.

#### 2.3.3. Perceived vulnerability to disease as a mediator in the relationship between perceived social status and PTSD symptoms

**Figure 1:**
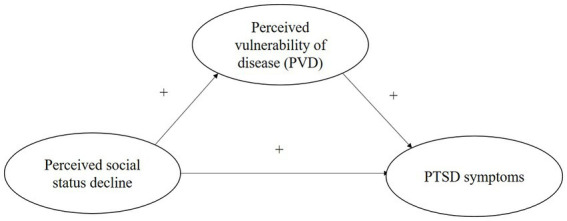
Conceptual framework.

At the same time, previous studies have proven the effects of perceived vulnerability to the disease on the development of various mental health diseases and symptoms, including traumatic stress reactions, which could develop into chronic PTSD (10, 26). Perceived vulnerability to disease refers to the sense that it is easy for oneself to come into contact with infectious diseases and a feeling of aversion to viruses, which may result in an increase in multiple health protection behaviors. Although having a sense of vulnerability to coronavirus infection during the pandemic contributes to the adoption of more self-protective behaviors, individuals with a strong perception of vulnerability to COVID-19 may have a lower sense of control or safety, which further leads to anxiety and traumatic stress reactions (26). As claimed by the transactional model of stress (8), an individual’s perception of threatening circumstances is more strongly linked to distress than the objective event itself. The COVID-19 pandemic has caused numerous infection cases and deaths since its outbreak, creating tremendous panic and worry in public regarding health and safety. Furthermore, with continual exposure to COVID-19-related news and stressful content through the media and other social networking sources, an increasing degree of COVID-19-related worries and distress has been found in the general population [e.g., (25, 45, 46)]. Consistent with the transactional model of stress, the cognitive model of PTSD (9) also suggests that psychological reactions to traumatic events might differ depending on how they are appraised (e.g., appraisals of danger lead to fear) and that the development of PTSD is more likely when individuals’ appraisals generate a “sense of serious current threat” (p. 320). Given the high transmission rate and mortality of the COVID-19 pandemic, it is reasonable to assume that it has generated a widespread sense of vulnerability to disease (26). Furthermore, as supported by the theory of the fundamental social cause, perceiving oneself as having lower social status generates higher risk perceptions, leading to more perceived vulnerability to disease (11). Considering the constant worries about personal and family health plus the insecurity in employability and related decline in social status, individuals with a higher perception of vulnerability to disease could encounter higher risks of developing PTSD or stress-related symptoms. Therefore, in this study, we also propose the following hypothesis:

*Hypothesis 2:* Perceived vulnerability to disease mediates the positive effects of a decline in perceived social status on the development of PTSD symptoms in people who were in Hubei during the first outbreak of the pandemic in 2020 (Figure 1).

### 2.4. The current study

As the region in which the coronavirus was first discovered in 2019, Wuhan and the whole Hubei Province of China could be considered the most severely affected regions worldwide at the beginning of the pandemic. In Hubei Province, where the data of this study were collected, social distancing measures and quarantine policies were adopted from January 23 to April 8, 2020, in most parts of the province. An immense number of employees had their employment contracts temporarily terminated or suspended due to the large-scale suspension and closure of numerous industrial activities for two and a half months (26). Furthermore, the instability of the infection rate and anti-pandemic measures caused additional uncertainty for workers regarding their employment security, which is closely related to their perception of social status. To investigate the potential detrimental consequences of these experiences, the study gathered data from Hubei 2 months after the release from confinement. For our method, we tried to control confounding variables via the counterfactual framework of propensity score matching, which is widely used to overcome the shortage of cross-sectional data in the fields of epidemiology, health services research, economics, and social sciences (47). This study had two major aims: first, to analyze the relationship between perceived social status decline and the prevalence of PTSD symptoms in the adult population in Hubei. Second, we investigated the potential mediating effect of perceived vulnerability to disease on the relationship between the two variables mentioned before. Therefore, our goal was to conduct an initial analysis of how subjective socioeconomic factors can influence people’s psychological well-being in the context of a prolonged public health emergency in the region where the pandemic originated.

## 3. Methods

### 3.1. Sampling

As the cities where the coronavirus outbreak began, Wuhan and other cities in Hubei Province were locked down from January 23 to April 8. The present work is based on an original study conducted in Hubei in June 2020—the crucial period of psychological adjustment for residents—by the School of Sociology, Central China Normal University in China. Out of the infection risk and prevention requirements, data were collected by an online questionnaire during the lockdown time, including several modules on mental health, family relationships, and social interaction. In the thematic modules involved in this study, a total of 3,465 valid participants aged above 16 responded to it. The sample comprised 52.90% males; the average age was 31.81 years; and 26.93% lived in Wuhan City. The research received ethical approval from the School of Sociology ethics committee at Central China Normal University in China.

We distributed electronic questionnaires through the trade union platform of Hubei, targeting a total of 14 million workers (including migrant workers) across the entire province. To reduce sampling bias, we initially focused the survey on workers aged 16 and above residing in county-level or higher cities within Hubei province. We implemented a filtering prompt in the first question of the questionnaire. Additionally, we provided a 100% chance of monetary incentive to encourage widespread survey sharing. We excluded samples with response of less than 5 min and samples that exhibited logical inconsistencies. Furthermore, we employed measures such as IP address identification and restrictions on accounts to minimize duplicate submissions. Lastly, to obtain a representative sample, we appropriately weighted the data using population statistics provided by the Hubei Provincial Federation of Trade Unions.

### 3.2. Measurement

#### 3.2.1. PTSD level

The dependent variable was the PTSD level. It was measured by The Impact of Event Scale–Revised (IES-R), which is based on three clusters of symptoms identified in the *Diagnostic and Statistical Manual of Mental Disorders*, to assess subjective distress caused by traumatic events. Participants were asked to rate their distress status from never (score 0) to all the time (score 4) with 22 items. In addition to the mean PTSD level applied in the models, we also report dichotomous data in Appendix I according to the cutoff of 1.5 suggested by Creamer et al. (48). The overall Cronbach’s coefficient of the scale was 0.980.

#### 3.2.2. Perceived social status decreases

The independent variable was perceived social status decrease. The participants were asked to what extent COVID-19 inflected their social status in the survey. We based the response and classified the participants into two mutually exclusive types: the decrease group (coded as 1) and the control group (perceived social status increased or remained the same, coded as 0).

#### 3.2.3. Mediator

In addition, we used the perceived vulnerability to disease as our mediator, which was measured by The Perceived Vulnerability to Disease Questionnaire (49). It is a widely used 15-item seven-point scale, ranging from strongly disagree (1) to strongly agree (5). It assesses one’s beliefs about personal susceptibility to and emotional discomfort associated with a potential contagion from infectious diseases. To enhance the cross-cultural adaptability, we deleted the fourth item (“I do not like to write with a pencil someone else has obviously chewed on.”) and kept 14 items. The goodness of fit test showed that the population follows the distribution [χ^2^(58) = 2012.008, SRMR = 0.12, CFI = 0.94, TLI = 0.91, RMSEA = 0.09]. The overall Cronbach’s coefficient of the scale was 0.930.

#### 3.2.4. Covariates

Based on the literature review, we found potentially available explanatory factors for PTSD perception. We included personal factors, infectious-related factors, and social factors, which are presented in Table 1.

**Table 1:** Covariate meanings and measurements.

Covariates	Meanings and measurements
*Personal factors*	
Age	Age as of 2022.
Gender	Male and female.
Education	The number of years of education a person completed.
Party	Whether one was a Party member.
Household registration	It was categorized into four level (countryside, town, rural–urban fringe, and urban areas) depending on the distance to city center.
Job status	Job status in the last 3 months.
Income	The average monthly income of family since 2020 (16 grades).
Sleep health	The product of sleep time (hours) and sleep quality, and sleep quality was rated by participants from very bad (1) to very good (4).
*Infectious-related factors*	
Perceived income decrease	The extent of COVID-19 inflected on family income.
Critical negative events	Whether one had COVID-19 cases (close-contact cases, suspected cases, confirmed cases, or death cases) in the family.
Exposure to epidemic information	The average amount of time participants had spent searching and reading epidemic information since the lockdown.
*Social factors*	
Interpersonal relationship (with family)	The frequency of quarrel with child/spouse during the pandemic, from no at all (1) to very frequent (3).
Interpersonal relationship (with epidemic prevention personnel)	Whether one had conflicts with epidemic prevention personnel.
Strictness of lockdown (subject)	Subjective feeling to lockdown policy, from no at all (1) to very strict (5).
Strictness of lockdown (object)	Objective frequency of going out, from no at all (1) to very frequent (5).
Encounter of Hubei citizens	The number of following things participants have encountered: (a) See comments on the internet or in chat groups that discriminate against or curse Hubei/Wuhan citizens; (b) Be refused to accept by local government and communities when returning hometown during the Spring Festival; (c) Be excluded when travelling, such as not allowed to stay at hotels; (d) Be ostracized and attacked by relatives and neighbors when returning hometown during the Spring Festival; (e) Be rejected by boss because of being Wuhan/Hubei citizens when returning to work; and (f) Be shunned and ostracized by colleagues because of being Wuhan/Hubei citizens after returning to work.
Fixed: city	A categorical variable, including Wuhan city, other cities in Hubei province, Hubei/Anhui/Henan provinces near Hubei, other provinces in China.

### 3.3. Analytical strategy

We followed a two-step analytical strategy to empirically examine the association between the decrease group and the control group. In the first step, we performed a propensity score analysis to control for potential selection bias. We used a developed package—teffects psmatch—available in Stata 17.0 to estimate the average treatment effect on the treated (ATET). We adopted a 1:1 matching strategy with replacement, estimated the p score by a logit model, and set the default caliper. Only the sample in common support was matched. In the second step, we estimated an ordinary least-squares linear regression model and multiple linear regression using social status decrease as the key response. The goal is to understand the different effects of whether social status decreased or not on the probability of PTSD levels among citizens after adjusting for a set of 18 covariables. Model 1 was our baseline model. Based on Model 1, Model 2 added demographic covariates, and Model 3 added all covariates. The matched columns show the compared result of estimates after applying sample weight depending on the number of matching times generated during matching. Finally, we checked the possibility of PVD as a mediator of the model.

## 4. Result

### 4.1. Descriptive statistics

Descriptive statistics are presented in Appendix I to summarize the sample’s characteristics and examine the variables’ distributions. Overall, 21.53% of participants’ social status decreased during the lockdown, whereas 78.47% increased or remained the same. The average PVD level was approximately 2.95. Nearly one-quarter of the sample had PTSD symptoms; the average education year was 13.95 years; 25.63% were Party members; 26.93% were Wuhan citizens in our sample, while 51.66% lived in the countryside far away from the city center; and 6.84% did not have jobs in the 3 months before our survey. Only 5.97% of respondents did not have conflicts with epidemic protection personnel; almost half of them thought the lockdown policy was stringent, and 64.76% did not have the opportunity to leave their homes. A total of 6.84% had COVID-19 cases in their family. On average, our respondents spent 2.52 h searching or reading COVID-19 information; each citizen encountered 1.4 negative incidents.

We also compared the characteristics between the treatment group (decrease group) and the control group. The mean PTSD level in the treatment group was significantly higher than the control group, both before and after matching. Before matching, the likelihood of being in the decrease group was greater for participants who were non-Party members, living in urban areas, with perceived income decreases and frequent quarreling with families compared with those in the control group. The likelihood of being in the decrease group was smaller for participants who lived in the countryside, had no COVID-19 cases in their families, and lived in Wuhan than for those in the control group. On average, participants in the control group had lower PTSD levels and healthier sleep and encountered fewer negative things in life. Before matching, the likelihood of being in the decrease group was greater for participants who were male, non-Party members, living in the countryside, perceiving an income decline, having a worse relationship with family and epidemic protection personnel, feeling that the lockdown policy was strict, having worse sleep health (below average), living in other cities in Hubei, above average reading of epidemic information, and encountering more negative things in life compared with those in the control group.

### 4.2. Multivariate results

**Figure 2:**
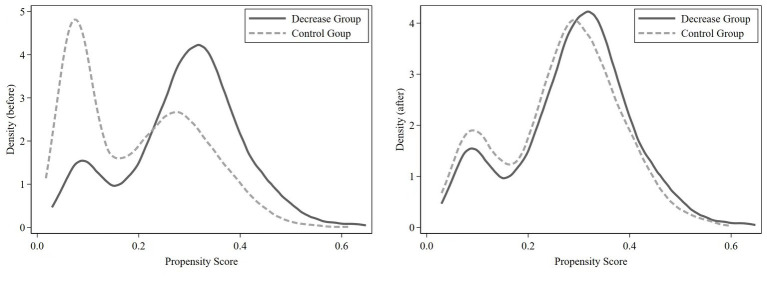
Kernel density estimate before and after matching.

Before estimating ATET, we checked the quality of PSM. We conducted paired t-tests with the propensity-score-matched groups. The results showed that the difference between groups was insignificant after matching and excluding the treatment variable (see the compared *p* value in Appendix I). We also found that the normalized bias of most variables in the matched groups was less than 10%, and most *t*-tests did not reject the null hypothesis that there was no systematic difference between the treatment group and the control group (Table 2). In addition, only 26 observations are off common support, which means we lost a few samples during matching. Figure 2 shows the comparison of the kernel density estimate between the treatment group and the control group, directly showing the good quality of matching.

**Table 2:** Balancing hypothesis test showing the variables’ characteristics before and after matching.

Variables	Unmatched	Mean		Bias (%)	*t*-Value	*p*-Value
	Matched	Treated group	Control group			
Gender	U	0.550	0.523	5.3	1.27	0.203
	M	0.551	0.561	−2.2	−0.42	0.676
Age	U	31.582	31.870	−3.1	−0.74	0.458
	M	31.509	31.698	−2	−0.4	0.692
Education	U	13.588	14.047	−15.7	−3.87	0.000
	M	13.588	13.571	0.6	0.12	0.907
Party	U	0.232	0.263	−7.2	−1.72	0.085
	M	0.231	0.221	2.2	0.43	0.664
Household registration	U	2.865	3.091	−19.1	−4.72	0.000
	M	2.872	2.848	2.1	0.39	0.699
Job status	U	0.914	0.914	0.2	0.05	0.956
	M	0.914	0.927	−4.8	−0.96	0.338
Income	U	4.400	5.271	−31.6	−7.25	0.000
	M	4.408	4.377	1.1	0.25	0.804
Perceived income decline	U	0.851	0.551	69.5	15.45	0.000
	M	0.850	0.848	0.6	0.14	0.885
PVD	U	2.981	2.936	13.7	3.32	0.001
	M	2.975	2.983	−2.5	−0.49	0.628
Quarrel with family	U	1.814	1.644	23.9	5.95	0.000
	M	1.807	1.757	7	1.33	0.182
Conflict with personnel	U	0.932	0.943	−4.5	−1.12	0.262
	M	0.935	0.930	2.2	0.41	0.679
COVID-19 cases	U	0.075	0.067	3.3	0.81	0.415
	M	0.073	0.067	2.1	0.41	0.684
Strictness of lockdown policy	U	4.245	4.348	−10.6	−2.66	0.008
	M	4.246	4.250	−0.4	−0.08	0.937
Frequency of going out	U	1.414	1.449	−5.2	−1.23	0.218
	M	1.413	1.432	−2.8	−0.55	0.58
City	U	1.310	1.265	4.1	0.99	0.325
	M	1.313	1.216	8.9	1.74	0.082
Sleep health	U	19.862	21.609	−21.6	−5.28	0.000
	M	19.918	19.980	−0.8	−0.15	0.882
Epidemic information	U	2.580	2.500	4.6	1.12	0.263
	M	2.563	2.621	−3.3	−0.65	0.519
Encounters	U	1.635	1.330	20.5	5.01	0.000
	M	1.614	1.646	−2.2	−0.4	0.687

U, unmatched; M, matched.

Table 3 provides the results of the matching. The level of PTSD in the treatment group was 0.266 higher than that in the control group on average, which means that a social status decrease could increase the PTSD level by 0.266 on average (*p* = 0.000, SD = 0.053).

**Table 3:** Average treatment effect of social status decline on PTSD level.

	Coefficient	AI robust std. err.	*z*	*P* > *z*	[95% confidence interval]	
ATET	0.226	0.053	4.22	0.000	0.121	0.330

We tested for homoscedasticity with the Breusch-Pagan/Cook–Weisberg test, which indicated OLS robust estimations in all cases except matched Model 3 (matched) in order to control for heteroskedasticity. We checked potential multicollinearity issues by computing the Variance Inflation Factor (VIF). Results for mean VIF range between 1.00 and 1.17, and all individual VIFs are well below 1.5. This is far below values that would suggest any multicollinearity issue being relevant. To account of potential correlation across observations for districts within the same cooperative arrangement, we cluster our estimations by city unit. The Durbin–Watson statistics of our models indicate no autocorrelation problems in unmatched models. After introducing propensity score weighting, matched models unavoidably exhibit a certain degree of autocorrelation. The Shapiro–Wilk test showed that some variables were not distributed normally. Therefore, we used the robust regression method to test the structural models.

Table 4 presents estimates of the average effect of social status decrease on PTSD levels (standard errors in parentheses) with different specifications. Model 1 and Model 2 seemed unable to support our hypothesis among the matched sample. The results seem relatively robust, with positive coefficient estimates, which remain significant after adding all covariates (Model 3, $\beta_{\mathrm{matched}}$ = 0.185***, *R*^2^ = 0.259). The models suggest that a greater decrease in social status is correlated with a worse degree of PTSD symptoms. Meanwhile, the results also show that the higher income group, people susceptible to disease, people quarreling frequently, and people with more negative encounters reported higher PTSD levels. In contrast, sleep quality was significantly negatively correlated with PTSD after adjustment.

**Table 4:** Effect of social status decline on PTSD level.

	Model 1		Model 2		Model 3	
	Unmatched	Matched	Unmatched	Matched	Unmatched	Matched
Social status decline	0.338^***^ (0.0371)	0.0631 (0.0539)	0.314^***^ (0.0358)	0.0587 (0.0570)	0.208^***^ (0.0256)	0.185^**^ (0.0477)
Gender			−0.0159 (0.0399)	0.0827 (0.0816)	0.0173 (0.0263)	0.100 (0.0591)
Age			0.00218 (0.00193)	−0.000139 (0.00233)	0.000863 (0.00200)	0.00184 (0.00152)
Education			0.00271 (0.00969)	−0.0200 (0.0123)	0.00544 (0.00821)	−0.0128^*^ (0.00523)
Party			0.0410 (0.0484)	0.0428 (0.0473)	−0.00479 (0.0340)	−0.0516 (0.0694)
Household registration			0.0523^**^ (0.0126)	0.0611 (0.0386)	0.0266^*^ (0.00902)	0.0461 (0.0263)
Job status			0.0785 (0.0492)	−0.0733 (0.0600)	0.0137 (0.0715)	−0.0951^**^ (0.0242)
Income			0.0142 (0.00779)	0.0508^*^ (0.0208)	0.0208^**^ (0.00632)	0.0527^**^ (0.00973)
Perceived income decline			0.172^***^ (0.0213)	0.212 (0.119)	0.0943^**^ (0.0234)	0.209 (0.0991)
PVD					0.846^***^ (0.0534)	1.014^***^ (0.0953)
Quarrel with family					0.0945^**^ (0.0257)	0.151^***^ (0.00762)
Conflict with personnel					−0.0949 (0.106)	0.109 (0.147)
COVID-19 cases					0.442^***^ (0.0341)	0.124 (0.167)
Strictness of lockdown policy					−0.00567 (0.0216)	−0.0236 (0.0737)
Frequency of going out					0.0124 (0.0255)	−0.00590 (0.0997)
Sleep health					−0.0213^***^ (0.00134)	−0.0163^*^ (0.00516)
Epidemic information					0.0229^**^ (0.00541)	0.0291 (0.0324)
Encounters					0.0983^***^ (0.00713)	0.139^***^ (0.0175)
_cons	0.839^***^ (0.0608)	0.951^***^ (0.0813)	0.325^**^ (0.0701)	0.663^*^ (0.254)	−1.829^***^ (0.269)	−2.698^***^ (0.355)
VIF	1.00	1.00	1.13	1.14	1.12	1.17
B-P/C-W Test *p*	0.012**	0.069*	0.039**	0.281	0.000***	0.000***
Durbin–Watson	1.889	0.706	1.898	0.695	1.940	0.717
*N*	3,465	3,439	3,465	3,439	3,465	3,439
*R* ^2^	0.022	0.001	0.037	0.041	0.232	0.259

Robust standard error are clustered at city level. Standard errors in parentheses. ^*^p < 0.1, ^**^p < 0.05, ^***^p < 0.01.

### 4.3. Mediating effect

Given the relatively higher performance of PVD in Model 3 ( $\beta_{\mathrm{matched}}\;$ = 1.014***, SD = 0.00973) and the theoretical basis, we checked the possibility of PVD as a mediator of the model. We used bootstrapping via Stata 17.0 to test for potential mediating effects. We adopted the recommended 95% confidence intervals (the bias-corrected percentile method) and used 2,000 bootstrap samples (50). Table 5 shows that PVD acted as a partial mediator, buffering the effects of social status decrease on PTSD levels. The indirect effect (0.039***) and the direct effect (0.207***) were statistically significant. Thus, Hypothesis 2 was supported.

**Table 5:** Mediating effect of PVD.

	Point estimate	Product of coefficient		Bootstrap 2,000 times, 95% CI (Bias-corrected percentile method)		
		SE	*z*	Lower	Upper	*p* value
Direct effect	0.207	0.036	5.72	0.134	0.276	0.000
Indirect effect	0.039	0.012	3.32	0.017	0.061	0.001

## 5. Discussion

The main goal of our study was to examine an initial analysis of how socioeconomic factors can influence people’s psychological well-being in the context of a prolonged public health emergency in the region where the pandemic originated. Thus, we analyze the relationship between perceived social status and the prevalence of PTSD symptoms mediated by perceived vulnerability to disease in the adult population in Hubei Province. We conducted an online questionnaire survey (*N* = 3,285) in Wuhan in June 2020 using multiple linear regression and propensity score matching analysis strategy. The study found that a decrease in perceived social status would lead to an increase in their PTSD levels compared to people with a constant perceived social status, and each decrease in the perceived unit of social status increases the level of PTSD by approximately 0.1 to 0.4 units. Perceived vulnerability to disease plays a partial mediating role in the positive relationship between perceived social status decline and an increase in PTSD. Although the indirect effect (0.039***) and the direct effect (0.207***) were statistically significant, we failed to capture the solid mediating effect of perceived vulnerability to disease.

### 5.1. The decisive role of perceived social status in mental health prediction

It is worth mentioning that the present research departs from those studies that focus on objective conditions. In the context of the COVID-19 pandemic, the decline in socioeconomic status is seen as a threat. The widespread use of social isolation policies, a decline in economic income, and occupational instability have all brought about negative mental and physical outcomes (31). However, existing studies have overlooked that perceived threats are a more direct factor causing PTSD compared to objective conditions. There is evidence to suggest that the relationship between subjective social status and mental health complies with the same reverse gradient found using objective social status indicators (51). We controlled for the variables closest to SES indicators (education, income, perceived income) and still obtained evidence of the positive impact of perceived social status decline on PTSD. This suggests that subjective social status may reflect unique aspects of socioeconomic status and may be more powerful in determining certain health outcomes than traditional SES measures.

In addition, in terms of the decisive prediction of subjective social status on mental health, the present finding is reasonable and consistent with previous research. Specifically, the conclusion further confirms the decisive role of subjective social status in health prediction (39). Compared with objective socioeconomic status, subjective socioeconomic status perception has a stronger effect on people’s well-being (19). Alcover et al. (41) found in a survey of adults in Chile from March to April 2020 that job insecurity and financial threats are associated with a decline in people’s general mental health. Especially in countries with collectivist cultures, people perceive socioeconomic status through social relations and social support, which has a more direct predictive effect on their mental health (52). The current conclusion is also closely related to the cognitive model of PTSD (9), in which the negative evaluation and memory of traumatic events have an impact on sustained PTSD. After a stressful event occurs, the focus is not on the event itself but on the negative evaluation of and sense of threat from the event. The cognitive model of PTSD (9) emphasizes the importance of subjective appraisal of a traumatic event when assessing the psychological impact of a trauma or stress. Subjective perceptions of a threat do not necessarily match the degree of threat indicated by more objective criteria and living conditions, and perceptions of threat are in fact more important in determining levels of distress.

### 5.2. Loss of indicator sensitivity of perceived vulnerability to disease for predicting PTSD

In terms of unexpected results, surprisingly, we found weak evidence for the mediating effects of perceived vulnerability to disease. Hypothesis 2 predicted that perceived vulnerability to disease mediates the positive relationship between a perceived decline in social status and PTSD. As shown in Table 5, perceived vulnerability to disease acted as a partial mediator, buffering the effects of perceived social status decrease on the level of PTSD symptoms. The indirect effect (0.039***) and the direct effect (0.207***) were statistically significant. Although Hypothesis 2 was supported, we failed to capture the strong mediating effect of perceived vulnerability to disease. Previous studies have proven the effects of perceived vulnerability to the disease on the development of various mental health diseases and symptoms, including traumatic stress reactions, which could develop into chronic PTSD (26). However, when comparing our results to those of older studies, it must be pointed out that the decisive role of subjective social status in mental health prediction may be the reason for this deviation, and the specific explanation is as follows.

First, this may be due to the high threat of COVID-19 to the maintenance of self-status, leading to the loss of indicator sensitivity of perceived vulnerability to disease for predicting PTSD. At the beginning of the COVID-19 pandemic, although people’s objective socioeconomic status has not changed, their feelings may not be the same. The impact of a decline in perceived social status on mental health typically occurs in elderly individuals, ethnic minorities, and immigrant groups (52–55). Green’s (52) study showed that compared to Hispanic immigrants who have immigrated to the United States for less than 3 years, immigrants who have resided in the US for more than 3 years have higher economic income, but their physical and mental health levels are worse. This is because the late-arriving group has never experienced a decline in socioeconomic status in their original residence. However, when they came to the United States, the perceived pressure of socioeconomic status decline led to their physical health level decline. Puerto Rican ethnic minority groups have also shown negative effects of reduced perceived social status on mental health (53). Research on the mental health of elderly people directly suggests a correlation between their perceived decline in social status and social acceptance (54, 55). Although our survey controlled for age, income, education level, and perceived income level, consistent results were obtained. In stress crisis events, adults experience a decrease in perceived social status, leading to an increase in their PTSD levels.

Furthermore, discrimination and stigmatization have a more direct impact on their mental health than perceived vulnerability to disease. The common view is that the outbreak of pandemic diseases may also have given rise to stigmatizing factors such as fear of isolation, racism, discrimination, and marginalization with all its social and economic ramifications (56). After strict quarantine policies, the number of infections reported every day gradually decreased after reaching its peak until it clears, and people believe that the actual infection range is controllable and traceable. Compared to the damage and harm caused by infectious diseases, the impact of discrimination experienced and heard by people had not disappeared since the release from quarantine (April 8, 2020) until the time of our investigation (June 2020). It is worth noting that the outbreak of the pandemic occurred during the Chinese New Year, and the 40-day “Spring Festival Movement” is an annual peak period of population mobility. Even if it was affected by the pandemic, the flow of 1.480 billion people is still a remarkable number (57). In view of the high transmission rate and high mortality rate of the COVID-19 pandemic, it is reasonable to believe that it has generated a wide range of disease susceptibilities (26), and mobility has exacerbated people’s panic. People who are considered to be at high risk of infection will suffer discrimination and stigmatization (31). Many people reported being discriminated against because of where they come from or currently lived during the panel outlet or whether they have been infected or have had close contact with confirmed cases (3). This has formed a tense and unacceptable atmosphere, bringing a sense of threat to the decline of their socioeconomic status, which is more urgent.

Finally, the perceived decline in social status at the beginning of the pandemic can directly predict perceived vulnerability to disease. When perceived job instability is assessed as a threat, the sense of stress, risk perception, and loss of control will increase, which will lead to enhanced perceived vulnerability to disease (26). Perceived vulnerability to the disease itself is caused by the perceived threat of social status decline. Therefore, regardless of whether it is mediated by perceived vulnerability to disease, PTSD is ultimately caused by the perceived threat of social status decline. Perceived vulnerability to disease partially mediates the relationship between perceived social status decline and the prevalence of PTSD symptoms, but the utility is not significant. This further confirms the decisive role of subjective social status in mental health prediction.

### 5.3. Practical implications

We contribute to the dialog on socioeconomic inequality by clarifying how perceived social status affects the prevalence of PTSD symptoms in the early days of the COVID-19 outbreak. Based on cognitive-relational theory, research has mainly been conducted from the perspective of perceptual evaluation. Our research extends this theory to the examination of socioeconomic status.

Furthermore, our findings have several practical implications. The conclusion reminds us that for individuals, a positive understanding of sudden crisis events can serve as a long-term resource to protect their mental health. Many studies have mentioned the positive role of supporting networks or resources in protecting individual mental health (11) and people’s sense of threat to events such as job instability, declining economic income, and loss of professional reputation (31, 41), which is the fundamental cause of PTSD. This reminds us that when public crisis events erupt, policymakers and social service providers need to apply event response techniques when intervening with individuals, starting from the trauma victim’s understanding of the event to solving the problem, and treating their PTSD or other mental trauma may be effective. During the pandemic, various interventions can be incorporated into positive psychological factors, including but not limited to helping people find a sense of meaning and coherence and utilizing self-compassion, gratitude, hope, and other personality strengths to cultivate positive and optimistic emotions (58).

More importantly, given the significant impact of perceived social status on the prevalence of PTSD symptoms in individuals, it is necessary to increase social support. There is established evidence that higher levels of social support predict higher perceived social status (52). It should be emphasized that intervention at the community level is more effective than intervention at the individual level, especially when people perceive themselves as belonging to a minority group (53). During the spread of the pandemic, at the community level, positive feedback from community workers and social service providers to residents who encounter difficulties is beneficial for protecting their perceived social status, which is effective and necessary. Specific measures can increase support for psychological counseling for community residents, as well as provide sufficient supply when they encounter social isolation, with special attention to forming support in relationships and social interactions. Given the high transmission rate of the pandemic, online network support is also a more suitable and convenient method. Through online technology, people’s social interactions are reconnected, which has been proven to have practical effects.

Especially, protection can be implemented through public policies to reduce people’s sense of discrimination and stigmatization. During the outbreak of the epidemic, quarantine is a common control measure. However, the widespread use of isolation of quarantine has brought widespread panic, acute stress disorder, anxiety, insomnia, and other adverse psychological symptoms (31). The author has personally experienced 14 days of strict centralized isolation, and suggested that the following key actions could be effective: first, maintain transparency of information, from the preparation before isolation to the action under surveillance during isolation, and during the period of home isolation after isolation, the government executives need to maintain full communication with relevant parties. The second is to ensure sufficient supply, basic water, food, and epidemic prevention supplies should be available at all times, and comfortable accommodation should be provided as much as possible to alleviate anxiety. The third is to establish a virtual support network, such as establishing centralized online communication groups for isolated populations and providing virtual space for mutual support. The fourth is to actively disseminate scientific epidemic prevention knowledge and protective information in news and public media, in order to alleviate discrimination against individuals under quarantine and residents in epidemic areas.

### 5.4. Limitations and future research

Taken together, our studies provide some compelling initial evidence for the significance of perceived social status for PTSD symptoms; however, further work is needed in several areas. First, this study was conducted in the early stages of the COVID-19 pandemic (June 2020), and its applicability to outbreaks is limited to the early stages. It is possible that the perceived social status response is caused by stress, and whether it has a long-term effect on PTSD as the pandemic eases and gradually disappears has not received attention. Second, the sample selection is based on the province where the pandemic broke out (Hubei Province, China), rather than the data collected nationwide. Our data was collected through an online questionnaire based on a trade union platform, which lacks representativeness compared to random sampling. However, we took various measures to reduce sampling bias. Our sample did not include an adequate number of confirmed COVID-19 cases as participants, and the research results should be interpreted with caution when applying them to confirmed cases. In addition, PSM relies on observational selection and cannot completely solve more general endogenous problems such as self-selection and missing variables. However, it constructs a counterfactual framework by reducing dependence on functional form settings. Weight adjustment generated in matching was also used to reduce bias as much as possible. Finally, our control variables did not consider the fluctuations in the market financial environment or the political conflicts and dynamics in the early stages of the epidemic. These variables are difficult to capture, and the impact of these variable relationships is unknown. Further research is suggested to be carried out among young people and elderly individuals in epidemic areas to observe the perceived long-term impact of socioeconomic status on the mental health of more vulnerable people.

## Data availability statement

The original contributions presented in the study are included in the article/Supplementary materials, further inquiries can be directed to the corresponding author.

## Ethics statement

The studies involving human participants were reviewed and approved by Institutional Review Board of Central China Normal University. The patients/participants provided their written informed consent to participate in this study.

## Author contributions

YW contributed to conception and design of the study. YW organized the database. SX performed the statistical analysis. YC, SX, and HL wrote sections of the manuscript. All authors contributed to the article and approved the submitted version.

## Funding

This work was supported by the National Social Science Foundation Project: Research on the Model of Revitalizing Rural Public Value through New Era Social Work Stations, China, Grant/Award Number: 22BSH128.

## Conflict of interest

The authors declare that the research was conducted in the absence of any commercial or financial relationships that could be construed as a potential conflict of interest.

## Publisher’s note

All claims expressed in this article are solely those of the authors and do not necessarily represent those of their affiliated organizations, or those of the publisher, the editors and the reviewers. Any product that may be evaluated in this article, or claim that may be made by its manufacturer, is not guaranteed or endorsed by the publisher.
