# Correction: A decline in perceived social status leads to post-traumatic stress disorder symptoms in adults half a year after the outbreak of the COVID-19 pandemic: consideration of the mediation effect of perceived vulnerability to disease

**DOI:** 10.3389/fpsyt.2025.1738903

**Published:** 2025-11-19

**Authors:** Yean Wang, Shuge Xu, Yue Chen, Haijuan Liu

**Affiliations:** 1 School of Social Development and Public Policy, Beijing Normal University, Beijing, China; 2 School of Sociology, Central China Normal University, Wuhan, China

**Keywords:** COVID-19, perceived social status, post-traumatic stress disorder, perceived vulnerability to disease, Hubei China, propensity score matching

Author Shuge Xu was erroneously assigned to affiliation "School of Sociology, Wuhan University, Wuhan, China". This affiliation has now been removed. The correct affiliation for Shuge Xu is "^1^School of Social Development and Public Policy, Beijing Normal University, Beijing, China".

The original version of this article has been updated.

