# Supplementary material for: A decline in perceived social status leads to post-traumatic stress disorder symptoms in adults half a year after the outbreak of the COVID-19 pandemic: consideration of the mediation effect of perceived vulnerability to disease
Source: Front Psychiatry. 2023 Jul 21;14:1217264. doi: 10.3389/fpsyt.2023.1217264 (PMC10402898; doi:10.3389/fpsyt.2023.1217264)
Supplement: Supplementary file 1 [file Table_1.docx]

Supplementary Material

A decline in perceived social status leads to post-traumatic stress disorder symptoms in adults half a year after the outbreak of the COVID-19 pandemic: consideration of the mediation effect of perceived vulnerability to disease

Wangwang Li, Shuge Xu, Yue Chen, Tianchen Zhang, Haijuan Liu^*^

*** Correspondence:** Haijuan Liu: [331260992@qq.com](mailto:331260992@qq.com)

*Appendix Ⅰ Descriptive Characteristics of Participants, according to perceived social status, before and after propensity score matching*

| Variables (range) | Total | | Unmatched | | | | | Matched | | | | |
| --- | --- | --- | --- | --- | --- | --- | --- | --- | --- | --- | --- | --- |
|  |  |  | 1=decrease | | 0=control | | P  value | 1=decrease | | 0=control | | P  value |
|  | Mean (%) | SD | Mean (%) | SD | Mean (%) | SD |  | Mean (%) | SD | Mean (%) | SD |  |
| PTSD level (0~4) | 0.91 | 0.94 | 1.18 | 0.98 | 0.84 | 0.92 | -0.338^***^ | 1.13 | 0.96 | 0.91 | 0.95 | -0.224^***^ |
| <1.5 | 73.16 |  | 61.93 |  | 76.24 |  |  | 63.03 |  | 72.89 |  |  |
| ≥1.5 | 26.84 |  | 38.07 |  | 23.76 |  |  | 36.97 |  | 27.11 |  |  |
| Gender |  |  |  |  |  |  | 0.203 |  |  |  |  | -0.008 |
| Female | 47.10 |  | 45.04 |  | 47.66 |  |  | 43.53 |  | 44.37 |  |  |
| Male | 52.90 |  | 54.96 |  | 52.34 |  |  | 56.47 |  | 55.63 |  |  |
| Age (16~70) | 31.81 | 9.39 | 31.58 | 9.13 | 31.87 | 9.46 | 0.288 | 31.43 | 9.10 | 31.39 | 9.18 | -0.038 |
| Education year | 13.95 | 2.87 | 13.59 | 3.03 | 14.05 | 2.82 | 0.458^***^ | 13.58 | 3.02 | 13.64 | 2.79 | 0.063 |
| Party |  |  |  |  |  |  | 0.031^*^ |  |  |  |  | 0.001 |
| Non-Party member | 74.37 |  | 76.81 |  | 73.70 |  |  | 76.13 |  | 76.06 |  |  |
| Party member | 25.63 |  | 23.19 |  | 26.30 |  |  | 23.87 |  | 23.94 |  |  |
| Household registration |  |  |  |  |  |  | 0.227^***^ |  |  |  |  | -0.01 |
| urban areas | 18.53 |  | 23.73 |  | 17.10 |  |  | 22.02 |  | 21.48 |  |  |
| rural-urban fringe | 10.36 |  | 10.99 |  | 10.19 |  |  | 10.92 |  | 12.32 |  |  |
| town | 19.45 |  | 20.38 |  | 19.20 |  |  | 21.01 |  | 20.77 |  |  |
| countryside | 51.66 |  | 44.91 |  | 53.51 |  |  | 46.05 |  | 45.42 |  |  |
| Job Status |  |  |  |  |  |  | -0.001 |  |  |  |  | 0.007 |
| No job | 8.63 |  | 8.58 |  | 8.64 |  |  | 8.40 |  | 7.75 |  |  |
| Had job | 91.37 |  | 91.42 |  | 91.36 |  |  | 91.60 |  | 92.25 |  |  |
| Perceived income change |  |  |  |  |  |  | -0.301^***^ |  |  |  |  | -0.007 |
| Increase/remain | 38.47 |  | 14.88 |  | 44.94 |  |  | 18.15 |  | 18.84 |  |  |
| Decrease | 61.53 |  | 85.12 |  | 55.06 |  |  | 81.85 |  | 81.16 |  |  |
| Income (1~16) | 5.08 | 2.93 | 4.40 | 2.48 | 5.27 | 3.02 | 0.872^***^ | 4.56 | 2.54 | 4.47 | 2.41 | -0.086 |
| PVD (1~5) | 2.95 | 0.33 | 2.98 | 0.32 | 2.94 | 0.33 | -0.045^***^ | 2.96 | 0.30 | 2.97 | 0.31 | 0.013 |
| Quarrel with family |  |  |  |  |  |  | -0.169^***^ |  |  |  |  | -0.052 |
| No at all | 44.99 |  | 38.74 |  | 46.71 |  |  | 41.01 |  | 41.90 |  |  |
| Ordinary | 41.93 |  | 41.15 |  | 42.15 |  |  | 41.18 |  | 44.54 |  |  |
| Very frequent | 13.07 |  | 20.11 |  | 11.14 |  |  | 17.82 |  | 13.56 |  |  |
| Conflict with personnel |  |  |  |  |  |  | 0.011 |  |  |  |  | -0.007 |
| No conflict | 5.97 |  | 6.84 |  | 5.74 |  |  | 6.55 |  | 7.22 |  |  |
| Had conflict | 94.03 |  | 93.16 |  | 94.26 |  |  | 93.45 |  | 92.78 |  |  |
| COVID-19 cases |  |  |  |  |  |  | -0.008 |  |  |  |  | -0.002 |
| No cases | 93.16 |  | 92.49 |  | 93.34 |  |  | 92.61 |  | 92.78 |  |  |
| Had cases | 6.84 |  | 7.51 |  | 6.66 |  |  | 7.39 |  | 7.22 |  |  |
| Strictness of lockdown policy |  |  |  |  |  |  | 0.103^***^ |  |  |  |  | 0.025 |
| Not at all | 2.54 |  | 3.62 |  | 2.24 |  |  | 3.87 |  | 2.46 |  |  |
| Not too much | 3.41 |  | 4.42 |  | 3.13 |  |  | 5.21 |  | 4.05 |  |  |
| Ordinary | 7.07 |  | 8.04 |  | 6.80 |  |  | 7.39 |  | 8.45 |  |  |
| Relatively | 32.90 |  | 31.64 |  | 33.25 |  |  | 31.26 |  | 35.74 |  |  |
| Very strict | 54.08 |  | 52.28 |  | 54.58 |  |  | 52.27 |  | 49.30 |  |  |
| Frequency of going out |  |  |  |  |  |  | 0.035 |  |  |  |  | 0.033 |
| Not at all | 64.76 |  | 65.95 |  | 64.44 |  |  | 66.55 |  | 65.32 |  |  |
| Not too much | 28.11 |  | 27.48 |  | 28.28 |  |  | 26.89 |  | 27.29 |  |  |
| Ordinary | 5.74 |  | 5.90 |  | 5.70 |  |  | 6.05 |  | 5.81 |  |  |
| Relatively | 0.98 |  | 0.54 |  | 1.10 |  |  | 0.34 |  | 1.23 |  |  |
| Very frequent | 0.40 |  | 0.13 |  | 0.48 |  |  | 0.17 |  | 0.35 |  |  |
| Sleep health (0~40) | 21.23 | 8.04 | 19.86 | 8.24 | 21.61 | 7.94 | 1.747^***^ | 20.21 | 8.30 | 20.17 | 7.82 | -0.041 |
| Epidemic information (0~10) | 2.52 | 1.74 | 2.58 | 1.74 | 2.50 | 1.74 | -0.081 | 2.49 | 1.69 | 2.59 | 1.74 | 0.099 |
| Encounters (0~6) | 1.40 | 1.48 | 1.64 | 1.53 | 1.33 | 1.46 | -0.306^***^ | 1.51 | 1.47 | 1.57 | 1.55 | 0.051 |
| City |  |  |  |  |  |  | -0.045 |  |  |  |  | -0.056 |
| Wuhan | 26.93 |  | 23.86 |  | 27.77 |  |  | 23.70 |  | 28.35 |  |  |
| Other cities in Hubei | 42.45 |  | 44.91 |  | 41.78 |  |  | 45.38 |  | 40.85 |  |  |
| Henan/Hunan/Anhui | 6.87 |  | 7.64 |  | 6.66 |  |  | 8.40 |  | 9.15 |  |  |
| Other Provinces | 23.75 |  | 23.59 |  | 23.80 |  |  | 22.52 |  | 21.65 |  |  |
|  |  |  |  |  |  |  |  |  |  |  |  |  |
| Total | 3465 |  | 746 |  | 2719 |  |  | 595 |  | 568 |  |  |

*Note**.* Wuhan is the capital city of Hubei Province. Henan/Hunan/Anhui are three provinces near Hubei. SD: standard deviation. ^*^ *p* < 0.1, ^**^ *p* < 0.05, ^***^ *p* < 0.01
